# Plasma Lipid Mediators Associate With Clinical Outcome After Successful Endovascular Thrombectomy in Patients With Acute Ischemic Stroke

**DOI:** 10.3389/fimmu.2022.917974

**Published:** 2022-07-04

**Authors:** Jiheng Hao, Yao Feng, Xin Xu, Long Li, Kun Yang, Gaolei Dai, Weiwei Gao, Meng Zhang, Yaming Fan, Tengkun Yin, Jiyue Wang, Bin Yang, Liqun Jiao, Liyong Zhang

**Affiliations:** ^1^ Department of Neurosurgery, Liaocheng People’s hospital, Liaocheng, China; ^2^ Department of Neurosurgery, Xuanwu Hospital, Capital Medical University, Beijing, China; ^3^ China International Neuroscience Institute (China-INI), Beijing, China; ^4^ Department of Evidence-based Medicine, Xuanwu Hospital, Capital Medical University, Beijing, China; ^5^ Department of Intervention, Liaocheng People’s hospital, Liaocheng, China; ^6^ Department of Neurology, Tianjin Huanhu Hospital, Tianjin, China; ^7^ Department of Interventional Neuroradiology, Xuanwu Hospital, Capital Medical Universit, Beijing, China

**Keywords:** acute ischemic stroke, endovascular thrombectomy (EVT), futile recanalization, cerebral ischemia-reperfusion injury, lipid mediator

## Abstract

**Background:**

Neuroinflammatory response contributes to early neurological deterioration (END) and unfavorable long-term functional outcome in patients with acute ischemic stroke (AIS) who recanalized successfully by endovascular thrombectomy (EVT), but there are no reliable biomarkers for their accurate prediction. Here, we sought to determine the temporal plasma profiles of the bioactive lipid mediators lipoxin A4 (LXA4), resolvin D1 (RvD1), and leukotriene B4 (LTB4) for their associations with clinical outcome.

**Methods:**

We quantified levels of LXA4, RvD1, and LTB4 in blood samples retrospectively and longitudinally collected from consecutive AIS patients who underwent complete angiographic recanalization by EVT at admission (pre-EVT) and 24 hrs post-EVT. The primary outcome was unfavorable long-term functional outcome, defined as a 90-day modified Rankin Scale score of 3-6. Secondary outcome was END, defined as an increase in National Institutes of Health Stroke Scale (NIHSS) score ≥4 points at 24 hrs post-EVT.

**Results:**

Eighty-one consecutive AIS patients and 20 healthy subjects were recruited for this study. Plasma levels of LXA4, RvD1, and LTB4 were significantly increased in post-EVT samples from AIS patients, as compared to those of healthy controls. END occurred in 17 (20.99%) patients, and 38 (46.91%) had unfavorable 90-day functional outcome. Multiple logistic regression analyses demonstrated that post-EVT levels of LXA4 (adjusted odd ratio [OR] 0.992, 95% confidence interval [CI] 0.987-0.998), ΔLXA4 (adjusted OR 0.995, 95% CI 0.991-0.999), LTB4 (adjusted OR 1.003, 95% CI 1.001-1.005), ΔLTB4 (adjusted OR 1.004, 95% CI 1.002-1.006), and post-EVT LXA4/LTB4 (adjusted OR 0.023, 95% CI 0.001-0.433) and RvD1/LTB4 (adjusted OR 0.196, 95% CI 0.057-0.682) ratios independently predicted END, and post-EVT LXA4 levels (adjusted OR 0.995, 95% CI 0.992-0.999), ΔLXA4 levels (adjusted OR 0.996, 95% CI 0.993-0.999), and post-EVT LXA4/LTB4 ratio (adjusted OR 0.285, 95% CI 0.096-0.845) independently predicted unfavorable 90-day functional outcome. These were validated using receiver operating characteristic curve analyses.

**Conclusions:**

Plasma lipid mediators measured 24 hrs post-EVT were independent predictors for early and long-term outcomes. Further studies are needed to determine their causal-effect relationship, and whether the imbalance between anti-inflammatory/pro-resolving and pro-inflammatory lipid mediators could be a potential adjunct therapeutic target.

## Introduction

Acute ischemic stroke (AIS) is a devastating cerebrovascular disease, and timely recanalization by intravascular thrombolysis and/or endovascular thrombectomy (EVT) is the primary therapeutic goal (1). Randomized controlled clinical trials and meta-analyses have established that EVT treatment for acute large vessel occlusion (LVO) within the proximal anterior circulation achieves >80% of near-complete or complete angiographic recanalization [modified thrombolysis in cerebral infarction score (mTICI) of 2b-3]. However, a proportion of AIS patients who were recanalized successfully develop early complications, including malignant brain edema, symptomatic hemorrhagic transformation, and secondary infarct growth, etc. Their symptoms and neurological deficits worsened within 24 hrs after EVT, known as early neurological deterioration (END) (2–4). Moreover, ~50% of successful recanalized AIS patients have a poor 3-month functional dependency [modified Rankin Scale (mRS) of 3-6], which is the condition that is termed as futile recanalization (5, 6). Accurate prediction of these early and long-term clinical outcomes may help to identify AIS patients suitable for EVT and optimize adjunct therapeutic strategies (4, 7).

Recanalization is often accompanied by cerebral ischemia-reperfusion (I/R) injury, which has been shown as a major cause of early complications, long-term neurological dysfunctions, or even death of AIS patients undergoing EVT (2, 3). Innate immunity and acute inflammatory response within the penumbra play important roles in the pathological progression of cerebral I/R injury. The regulation and execution of neuroinflammatory responses are mediated by inflammatory mediators, including vasoactive amines and peptides, lipid mediators, cytokines, chemokines, and complements (8–11). Novel biomarkers in neuroinflammation may therefore be of great value in predicting clinical outcomes in patients with AIS after EVT treatment. Recently, the lipid metabolism of the brain, especially that of polyunsaturated fatty acids (PUFAs) and their bioactive derivatives, has emerged as a key process of the neuroinflammation related to cerebral I/R injury (12). PUFAs are structural and functional constituents of cellular membranes that are enriched in cells of the central nervous system and that can be further divided into the omega-3 fatty acids such as docosahexaenoic acids (DHA) and eicosapentaenoic acids (EPA) that are found in fish oil and omega-6 fatty acids [e.g. arachidonic acid (AA) and alpha-linoleic acid (ALA)] that are often found in plant oil (12, 13). Bioactive lipid mediators are a large family of versatile molecules derived from PUFAs, and are involved in the initiation, progression, and resolution of inflammation. These lipid mediators include AA-derived prostaglandins (PGs; e.g., PGE2), leukotrienes (LTs; e.g., LTB4), and thromboxanes (TXs; e.g., TXA2), and an expanding list of newly discovered specialized pro-resolving mediators (SPMs). SPMs are mainly classed as AA-derived lipoxins (LXs; e.g., LXA4), DHA-derived resolvins (RvDs; e.g., RvD1), EPA-derived resolvins (RvEs; e.g., RvE1), neuroprotectins (NPs; e.g., NPD1), and maresins (MaRs; e.g., MaR1) (12, 13). These bioactive lipid mediators exert ambivalent role during inflammatory process. Previous studies using experimental model of cerebral I/R injury have shown that LTs, especially LTB4, induce an acute inflammatory response and aggravate brain damage, whereas SPMs dampen excessive inflammation and promote tissue repair shortly after the initiation of the acute inflammatory response (14–16). Hence, the balance of pro-inflammatory LTB4 and anti-inflammatory/pro-resolving SPMs during acute inflammation defines the duration and strength of the inflammatory response (17–19). To date, few studies have addressed the longitudinal changes of inflammation-related lipid mediators and their associations with clinical outcomes in AIS patients after EVT. To fill this critical knowledge gap, we enrolled patients with AIS who received successful recanalization by EVT to determine 1) the temporal profiles of plasma anti-inflammatory/pro-resolving LXA4 and RvD1, and pro-inflammatory LTB4 before (on admission) and 24 hrs after EVT, 2) the dynamic balance between SPMs and LTB4 (SPMs/LTB4 ratio), and 3) whether these lipid markers predict unfavorable early (END) and long-term functional outcomes.

## Material and Methods

### Study Population

We performed a retrospective analysis of plasma samples and clinical information of consecutive AIS patients with first-ever LVO admitted to Liaocheng People’s Hospital (Liaocheng, China) and Xuanwu Hospital, Capital Medical University (Beijing, China), and achieved complete angiographic recanalization by EVT. EVT was performed with the basis of available high-level national and international evidence and contemporary EVT techniques. The study period was May 22, 2019 to October 23, 2020. Inclusion criteria were EVT-treated AIS patients with 1) ≥18 years of age, 2) LVO of the anterior circulation [internal carotid artery (ICA), proximal middle Cerebral Artery (MCA), or tandem occlusion] identified by computed tomography (CT)-angiography, magnetic resonance angiography, or digital subtracted angiography, 3) groin puncture initiated within 6 hrs from the symptom onset (last known well), 4) mTICI of 3. Exclusion criteria were EVT-treated AIS patients with 1) posterior circulation stroke, 2) pre-stroke disability (mRS of 2-6), 3) unfavorable clinical outcomes occurred before sampling, 4) concurrent or recent infection, hematological, rheumatic, or autoimmune disorders, 5) on anti-inflammatory or immunosuppressant on admission, 6) active malignancy, 7) severe liver or kidney dysfunction, 8) incomplete clinical or follow-up data. In addition, during the same period, 20 age- and sex- matched healthy individuals who received physical examination in Liaocheng People’s Hospital and Xuanwu Hospital, Capital Medical University were recruited as controls to analyze temporal profile of plasma lipid mediators in EVT-treated AIS patients. This study was approved by the Ethics Committees of Liaocheng People’s Hospital and Xuanwu Hospital, Capital Medical University, and was carried out in accordance with the Helsinki declaration. All study participants or their legal representatives were informed of the study protocol and potential risks, and gave written consent for the study.

### Clinical Data Collection

We collected demographic characteristics (age and gender), vascular risk factors (hypertension, diabetes mellitus, dyslipidemia, coronary artery disease, atrial fibrillation, and current smoking), stroke severity on admission and 24 hrs post-EVT assessed using National Institutes of Health Stroke Scale (NIHSS) score, stroke location, stroke etiology stratified using Trial of Org 10,172 in acute stroke treatment (TOAST) classification system, procedural parameters (time from symptom onset to groin puncture, time from groin puncture to recanalization, number of maneuver passes, and intravenous thrombolysis prior to EVT), current lipid-lowering/anti-platelet/coagulation treatment, and the occurrence of symptomatic intracranial hemorrhage (sICH) diagnosed according to the criteria of the Heidelberg Bleeding Classification within 72 h post-EVT (20). The following clinicodemographic data were collected from the healthy controls: age, gender, vascular risk factors, and current lipid-lowering/anti-platelet/coagulation treatment. Functional outcome at 90 days post-EVT served as the primary study end-point and was assessed using mRS by telephone or outpatient review. Unfavorable outcome was defined as mRS of 3-6, while favorable outcome was defined as mRS of 0-2. The secondary end-point was END that was defined as an increase in NIHSS score ≥4 points from baseline to 24 hrs post-EVT (4, 21).

### Blood Collection and Lipid Mediator Measurements

Blood samples were drawn from the cubital veins of healthy subjects and AIS patients (on admission and 24 hrs post-EVT). Routine laboratory data of complete blood counts were collected, and the neutrophil/lymphocyte ratio (NLR) was calculated. For lipid marker measurement, sodium citrated blood samples (0.36% of final concentration) were centrifuged at 1,500 *g* for 15 min to collect the supernatant, which was then centrifuged at 13,000 *g* for 3 min to collect cell-free plasma and stored at -80°C until use. Levels of LXA4 (Cat. No. EK12146), RvD1 (Cat. No. EK11723), and LTB4 (Cat. No. EK10021) were quantified by enzyme-linked immunosorbent assay (ELISA) kits (SAB^®^ Signalway Antibody, Greenbelt, MD, USA) following the manufacturer’s instructions.

### Statistical Analysis

Statistical analysis was performed by SPSS 22.0 statistical software (IBM, Armonk, NY, USA), and statistical significance was set at two-tailed *p*<0.05. Data normality of distributions was performed with the Kolmogorov-Smirnov test. Continuous variables with a normal distribution were presented as the mean ± standard deviation (SD), and intergroup differences were analyzed using the independent-samples t-test. Non-normally distributed variables were presented as medians with interquartile range (IQR), and intergroup differences were analyzed using the Mann-Whitney U test. Categorical variables were presented as frequency and percentage, and intergroup differences were analyzed using the chi-square test (or Fisher’s exact test when the expected value was <5). To control the error rate under multiple testing, False Discovery Rate (FDR)-correction was conducted. To analyze dynamic changes of plasma levels of lipid mediators pre- and post-EVT, paired student’s t test (normally distributed continuous variables) or paired Wilcoxon signed-rank test (non-normally distributed continuous variables) was used. Spearman correlation coefficient was performed to correlate plasma lipid mediators with the 90-day mRS scores, and Pearson correlation coefficient was used to correlate plasma lipid mediators with NLR. The relationship between plasma lipid mediators and early (END) and long-term functional outcomes was analyzed by using the multivariate logistic regression analysis after adjustment for confounding variables (significant differences between subgroups). Results were expressed as adjusted odds ratio (OR) with the corresponding 95% confidence interval (CI). The receiver operating characteristic (ROC) curve and the area under the curve (AUC) were then used to determine the sensitivity, specificity, and optimal diagnostic values of these plasma lipid mediators to predict outcome.

## Results

### Baseline Characteristics of the Study Population

A total of 81 consecutive AIS patients with first-ever LVO of the anterior circulation who achieved complete angiographic recanalization by EVT (mean age 68.53 ± 10.63 years; 38 female) were enrolled in this study. We used the TOAST classification system to determine that 28 (34.57%) patients had cardioembolic stroke, 46 (56.79%) patients had large artery atherothrombotic stroke, and 14 (17.28%) patients had AIS for unknown reason. The median admission NIHSS score was 18 (IQR, 13-25.5). A bridging therapy with recombinant tissue-plasminogen activator (rt-PA) prior to EVT was performed in 49 (60.5%) patients. The current lipid-lowering, anti-platelet, and anti-coagulation treatment were observed in 15 (18.52%; atorvastatin: 12; rosuvastatin: 3), 18 (22.22%; aspirin: 16; clopidogrel: 2), and 3 (3.7%; heparin: 2; warfarin: 1) patients, respectively. Seven patients with AIS (8.64%) suffered sICH after recanalization. In addition, 20 matched healthy subjects (mean age 66.55 ± 11.58 years; 9 female) were enrolled and severed as baseline to analyze temporal profile of plasma lipid mediators in EVT-treated AIS patients. Among them, 3 (15%) underwent lipid-lowering therapy (all atorvastatin) and 3 (15%) received anti-platelet drugs (all aspirin) at the time of sample collecting. The demographic and clinical characteristics of the study population are summarized in **Table 1** and found to be comparable between AIS patients and healthy controls, except for the frequency rate of atrial fibrillation (6.67% vs. 35.8%, p=0.007, **Table 1**).

**Table 1:** Baseline characteristics of the study population.

Characteristic	Healthy Controls (n=20)	AIS Patients (n=81)	*p*	Early neurological deterioration			90-day functional outcome		
				No (n=64)	Yes (n=17)	*p*	mRS 0-2 (n=43)	mRS 3-6 (n=38)	*p*
Age, year mean ± SD or median (IQR)	66.55 ± 11.58	68.53 ± 10.63	0.465^a^	68 (62, 74)	75 (70, 78.5)	0.038^d^	65.58 ± 10.54	71.87 ± 9.83	0.007^a^
Gender, male/female, n	11/9	44/38	0.878^b^	38/26	5/12	0.028^b^	28/15	15/23	0.021^b^
Vascular Risk factors, n (%)									
Hypertension	7 (35%)	36 (44.4%)	0.444^b^	30 (46.27%)	6 (35.71%)	0.393^b^	16 (37.21%)	17 (44.74%)	0.491^b^
Diabetes	5 (25%)	16 (19.75%)	0.759^c^	12 (19.4%)	4 (21.43%)	0.734^c^	4 (9.3%)	11 (28.95%)	0.023^b^
Dyslipidemia	4 (20%)	10 (12.35%)	0.469^c^	9 (13.43%)	1 (7.14%)	0.68^c^	6 (13.95%)	3 (7.9%)	0.49^c^
Coronary artery disease	6 (30%)	23 (28.4%)	0.887^b^	17 (28.36%)	6 (28.57%)	0.549^c^	9 (20.93%)	13 (34.21%)	0.18^b^
Atrial fibrillation	1 (6.67%)	29 (35.8%)	0.007^b^	23 (37.31%)	6 (28.57%)	0.961^b^	13 (30.23%)	16 (42.11%)	0.266^b^
Smoking	5 (25%)	18 (22.2%)	0.772^c^	15 (23.88%)	3 (14.29%)	0.751^c^	9 (20.93%)	9 (23.68%)	0.766^b^
Admission NIHSS score, mean ± SD or median (IQR)	N/A	18 (13, 25.5)	–	19.8 ± 9.31	19.53 ± 7.8	0.914^a^	15 (12, 23)	23 (15, 26.75)	<0.001^d^
Stroke location, n (%)									
Internal carotid artery	N/A	22 (27.16%)	–	17 (26.86%)	5 (28.57%)	0.966^b^	12 (27.91%)	9 (23.68%)	0.901^b^
Middle cerebral artery	N/A	45 (55.56%)		36 (56.72%)	9 (50%)		24 (55.81%)	22 (57.9%)	
Tandem lesion	N/A	14 (17.28%)		11 (16.42%)	3 (21.43%)		7 (16.28%)	7 (18.42%)	
Stroke cause (TOAST), n (%)									
Cardioembolic	N/A	28 (34.57%)	–	21 (34.33%)	7 (35.71%)	0.772^b^	15 (34.88%)	13 (34.21%)	0.385^b^
Large artery atherosclerosis	N/A	46 (56.79%)		37 (56.72%)	9 (57.15%)		26 (60.47%)	20 (52.63%)	
Unknown	N/A	7 (8.64%)		6 (10.45%)	1 (7.14%)		2 (4.65%)	5 (13.16%)	
Procedural parameters									
Onset to groin puncture, min, median (IQR)	N/A	231 (157, 311.5)	–	229 (140.3, 294.3)	259 (196, 324.5)	0.144^d^	228 (157, 312)	249.5 (141, 313)	0.773^d^
Groin puncture to recanalization, min, median (IQR)	N/A	70 (49, 95.5)	–	70 (47.5, 94.75)	70 (54.5, 98.5)	808^d^	65 (39, 93)	72 (60, 98)	0.077^d^
Number of passes, 1/2/3/4/5, n	N/A	41/25/6/7/2	–	34/19/5/5/1	7/6/1/2/1	0.765^b^	25/12/2/4/0	16/13/4/3/2	0.339^b^
First pass, n (%)	N/A	41 (50.62%)	–	34 (52.24%)	7 (42.86%)	0.381^b^	25 (58.14%)	16 (42.11%)	0.15^b^
Prior IV rt-PA, n (%)	N/A	49 (60.5%)	–	41 (64.18%)	8 (42.86%)	0.202^b^	28 (65.12%)	21 (55.26%)	0.365^b^
Medications, n (%)									
Antiplatelet	3 (15%)	18 (22.22%)	0.557^c^	12 (17.91%)	6 (42.86%)	0.19^c^	7 (16.3%)	11 (28.95%)	0.171^b^
Anticoagulation	0 (0%)	3 (3.7%)	1.000^c^	2 (4.48%)	1 (0%)	0.512^c^	2 (4.65%)	1 (2.63%)	1.000^c^
Lipid-lowering	3 (15%)	15 (18.52%)	1.000^c^	12 (17.91%)	3 (21.43%)	1.000^c^	10 (23.26)	5 (13.16%)	0.243^b^
sICH, n (%)	N/A	7 (8.64%)	–	4 (5.97%)	3 (21.43%)	0.157^c^	2 (4.65%)	5 (13.16%)	0.244^c^
END, n (%)	N/A	17 (20.99%)	–	–	–	–	3 (6.98%)	14 (36.84)	0.002^c^

^a^Analysed by Independent sample t-test; ^b^Analysed by Chi-square test; ^c^Analysed by Fisher’s exact test. ^d^Analysed by Mann-Whitney U test; AIS, acute ischemic stroke; END, Early neurological deterioration. SD, standard deviation; NIHSS, National Institutes of Health Stroke Scale; N/A, not available; TOAST, Trial of Org 10172 in Acute Stroke Treatment; IV, intravenous; rt-PA, recombinant tissue plasminogen activator; mTICI, modified Treatment in Cerebral Infarction; sICH, symptomatic intracerebral hemorrhage.

### Temporal Profile of Plasma Lipid Mediators in EVT-Treated AIS Patients

**Figure 1:**
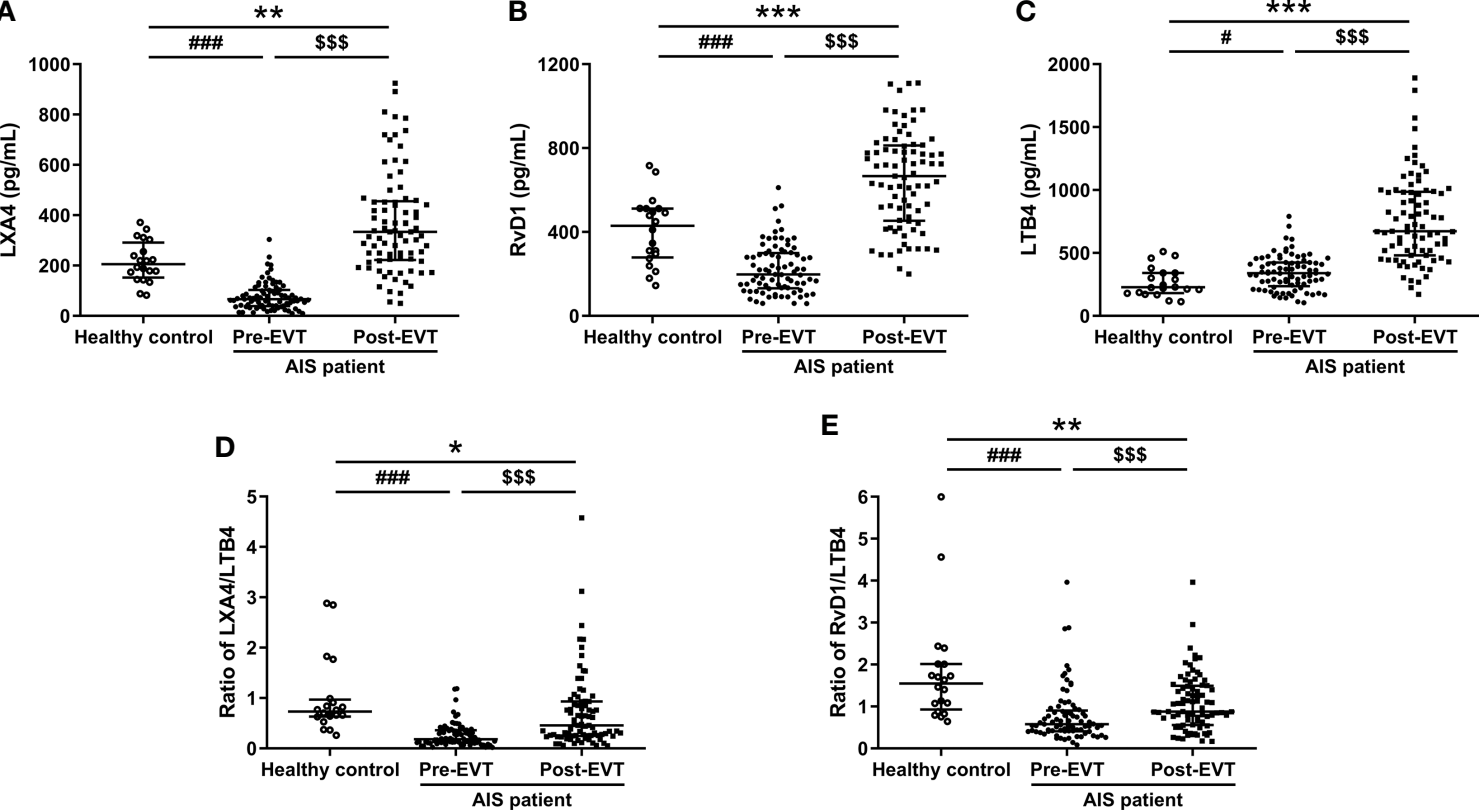
Plasma levels of LXA4 **(A)**, RvD1 **(B)**, LTB4 **(C)**, ratio of LXA4/LTB4 **(D)**, and ratio of RvD1/LTB4 **(E)** in healthy controls and EVT-treated AIS patients (pre- and 24 hrs post-EVT). Healthy controls were severed as baseline to analyze temporal profile of plasma lipid mediators in EVT-treated AIS patients. Data were presented as median and IQR. Differences between AIS patients and healthy controls were analyzed using the Mann-Whitney U test or independent-samples t-test (LTB4: healthy control vs. pre-EVT, RvD1: healthy control vs. post-EVT). The paired Wilcoxon signed-rank test was used to analyze dynamic changes of plasma levels of lipid mediators pre- and post-EVT. ^#^p<0.05 and ^###^p<0.001: healthy control vs. pre-EVT; *p<0.05, **p<0.01, and ***p<0.001: healthy control vs. post-EVT; ^$$$^p<0.001: pre-EVT vs. post-EVT.

As shown in **Figures 1A, B**, plasma levels of LXA4 and RvD1 were significantly lower in AIS patients on admission compared to healthy controls [LXA4: 66.38 (IQR 38.41-103.63) vs. 205.6 (IQR 151.7-291.37) pg/mL, p<0.001; RvD1: 197.16 (IQR 131.28-299.62) vs. 429.74 (IQR 278.75-511.35) pg/mL, p<0.001], but became higher 24 hrs post-EVT than their pre-EVT levels [LXA4: 333.74 (IQR 221.77-455.49) vs. 66.38 (IQR 38.41-103.63) pg/mL, p<0.001; RvD1: 666.3 (IQR 453.5-812.44) vs. 197.16 (IQR 131.28-299.62) pg/mL, p<0.001) and those of control subjects [LXA4: 333.74 (IQR 221.77-455.49) vs. 205.6 (IQR 151.7-291.37) pg/mL, p<0.001; RvD1: 666.3 (IQR 453.5-812.44) vs. 429.74 (IQR 278.75-511.35) pg/mL, p<0.001]. In contrast, pre-EVT LTB4 levels in AIS patients were significantly higher than those of healthy controls [339.12 (IQR 235.16-424.38) vs. 226.72 (IQR 181.3-341.34) pg/mL, p=0.027] and increased further 24 hrs post-EVT [672.9 (IQR 481.38-985.51) vs. 339.12 (IQR 235.16-423.38) pg/mL, p<0.001, **Figure 1C**]. The ratio of SPM to LTB4 has been previously used to define the balance between anti-inflammatory/pro-resolving and pro-inflammatory responses of the patients (19). As shown in **Figures 1D, E**, both pre-EVT LXA4/LTB4 ratio and RvD1/LTB4 ratio were significantly lower in AIS patients compared to healthy controls [LXA4/LTB4 ratio: 0.18 (IQR 0.12-0.36) vs. 0.73 (IQR 0.63-0.97) pg/mL, p<0.001; RvD1/LTB4 ratio: 0.58 (IQR 0.41-0.9) vs. 1.55 (IQR 0.93-2.01) pg/mL, p<0.001]. They were significantly increased in samples collected 24 hrs post-EVT [LXA4/LTB4 ratio: 0.46 (IQR 0.25-0.93) vs. 0.18 (IQR 0.12-0.36) pg/mL, p<0.001; RvD1/LTB4 ratio: 0.88 (IQR 0.56-1.49) vs. 0.58 (IQR 0.41-0.9) pg/mL, p<0.001], but remained lower than those of control subjects [LXA4/LTB4 ratio: 0.46 (IQR 0.25-0.93) vs. 0.73 (IQR 0.63-0.97) pg/mL, p=0.017; RvD1/LTB4 ratio: 0.88 (IQR 0.56-1.49) vs. 1.55 (IQR 0.93-2.01) pg/mL, p=0.004].

### Plasma Levels of Lipid Mediators and END

**Figure 2:**
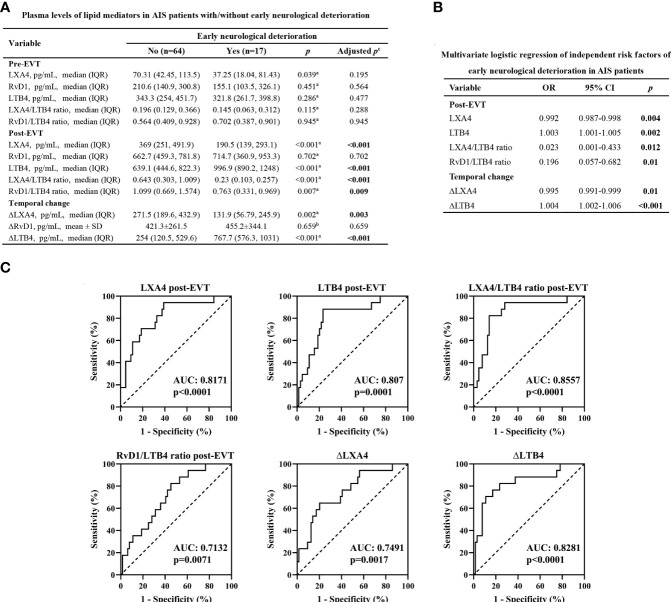
Plasma levels of lipid mediator parameters and END. **(A)** Plasma levels of lipid mediator parameters in EVT-treated AIS patients with or without END. ^a^Data were presented as median and IQR, and were analyzed by Mann-Whitney U test. ^b^Data were presented as mean ± SD, and were analyzed by independent-samples t-test. ^c^False Discovery Rate (FDR)-correction was conducted to control the error rate under multiple testing. **(B)** Multivariate logistic regression of independent risk factors of END. Confounding factors: age and gender. **(C)** Receiver operator characteristic (ROC) curve analyses for lipid mediator parameters to predict END after successful EVT in patients with AIS. OR, odds ratio; CI, confidence interval. AUC, area under the curve.

END occurred in 17 (20.99%) patients. Age (p=0.038) and proportion of female (p=0.028) were significantly higher in AIS patients who had END than in those who had no END (**Table 1**). In contrast, no statistical differences were observed between the two groups in vascular risk factors (including hypertension, diabetes, dyslipidemia, coronary artery disease, atrial fibrillation, and smoking), NIHSS score on admission, stroke location, stroke etiology, procedural parameters (including time from symptom onset to groin puncture, time from groin puncture to recanalization, number of passes, and intravenous thrombolysis prior to EVT), current medication treatment, and the occurrence of sICH (**Table 1**). As shown in **Figure 2A**, there were no significant differences in the lipid mediator parameters tested in samples collected on admission between the two groups (adjusted p>0.05). The post-EVT plasma LXA4 levels (adjusted p<0.001), the LXA4/LTB4 (adjusted p<0.001) and RvD1/LTB4 (adjusted p=0.009) ratios, and ΔLXA4 levels (adjusted p=0.003) were significantly lower, while those of post-EVT LTB4 levels (adjusted p<0.001) and ΔLTB4 (adjusted p<0.001) were significantly higher in samples from patients with END as compared to those with non-END. However, post-EVT levels of RvD1 (adjusted p=0.702) and ΔRvD1 (adjusted p=0.659) were comparable between the two groups. Multivariate logistic regression analyses revealed that post-EVT LXA4 levels (p=0.004), LTB4 levels (p=0.002), and the LXA4/LTB4 (p=0.012) and RvD1/LTB4 (p=0.01) ratios, as well as ΔLXA4 levels (p=0.01), and ΔLTB4 levels (p<0.001) were independent predictors of END after adjustment for confounding factors (age and gender; **Figure 2B**). To further evaluate the sensitivity, specificity, and predictive values of these variables for END, ROC curve was plotted (**Figure 2C**). The AUC for post-EVT LXA4 levels, LTB4 levels, the LXA4/LTB4 and RvD1/LTB4 ratios, ∆LXA4 levels, and ∆LTB4 levels were 0.8171 (95%CI: 0.7034-0.9308), 0.907 (95%CI: 0.6923-0.9217), 0.8557 (95%CI: 0.7506-0.9608), 0.7132 (95%CI: 0.5869-0.8396), 0.7491 (95%CI: 0.6192-0.879), and 0.8281 (95%CI: 0.7063-0.9499), respectively. Their corresponding optimal cutoff values were 333.8 pg/mL (sensitivity 60.94% and specificity 94.12%), 824.3 pg/mL (sensitivity 76.56% and specificity 88.24%), 0.258 (sensitivity 85.94% and specificity 82.35%), 1.065 (sensitivity 54.69% and specificity 82.35%), 166.4 pg/mL (sensitivity 76.69% and specificity 58.82%), and 655.3 pg/mL (sensitivity 89.06% and specificity 70.59%), respectively.

### Correlations of Plasma Levels of Lipid Mediators With 90-Day Functional Outcome

**Figure 3:**
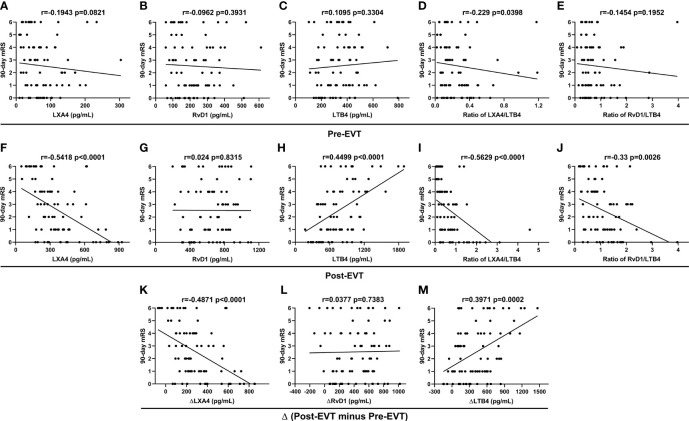
Spearman correlation coefficient analyses of correlation between plasma lipid mediator parameter levels in EVT-treated AIS patients and 90-day mRS scores.

Spearman correlation coefficient showed no correlation between the lipid mediator parameters measured on admission and 90-day mRS scores (p>0.05, **Figures 3A-C, E**), except for LXA4/LTB4 ratio (r=-0.229, p=0.0398, **Figure 3D**). Post-EVT LXA4 levels (r=-0.5418, p<0.001, **Figure 3F**), the ratios of LXA4-to-LTB4 (r=-0.5629, p<0.0001, **Figure 3I**) and RvD1-to-LTB4 (r=-0.33, p=0.0026, **Figure 3J**), and ΔLXA4 levels (r=-0.4871, p<0.001, **Figure 3K**) were inversely associated with the 90-day mRS scores, whereas levels of post-EVT LTB4 (r=0.4499, p<0.0001, **Figure 3H**) and ΔLTB4 (r=0.3971, p=0.0002, **Figure 3M**) were positive associated with 90-day mRS scores. There was no significant correlation between levels of post-EVT RvD1 (p=0.8315, **Figure 3G**) and ΔRvD1 (p=0.7383, **Figure 3L**) and 3-month mRS scores.

### Plasma Levels of Lipid Mediators and Unfavorable 90-Day Functional Outcome

**Figure 4:**
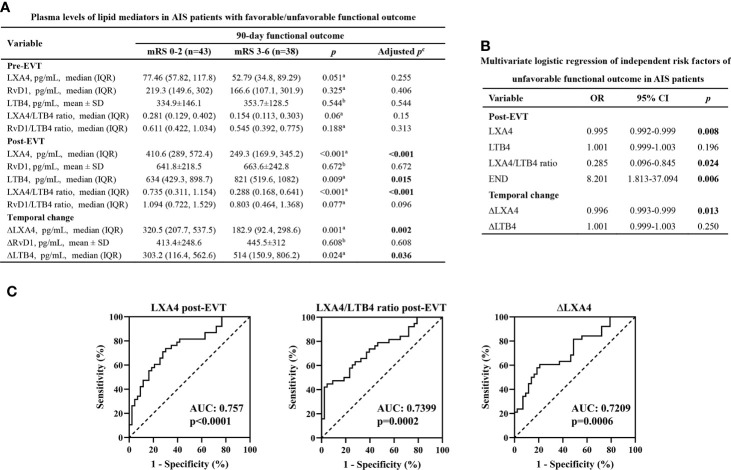
Plasma levels of lipid mediator parameters and 90-day functional outcome. **(A)** Plasma levels of lipid mediator parameters in EVT-treated AIS patients with favorable or unfavorable 90-day outcomes. ^a^Data were presented as median and IQR, and were analyzed by Mann-Whitney U test. ^b^Data were presented as mean ± SD, and were analyzed by independent-samples t-test. ^c^False Discovery Rate (FDR)-correction was conducted to control the error rate under multiple testing. **(B)** Multivariate logistic regression of independent risk factors of unfavorable 90-day outcome. Confounding factors: age, frequency rate of female, diabetes and END, and admission NIHSS scores. **(C)** Receiver operator characteristic (ROC) curve analyses for lipid mediator parameters to predict unfavorable 90-day outcome after successful EVT in patients with AIS. OR, odds ratio; CI, confidence interval. END, early neurological deterioration. AUC, area under the curve.

Among the 81 AIS patients, 43 (53.09%) had 90-day mRS of 0-2 (favorable outcome) and 38 (46.91%) had mRS of 3-6 (unfavorable outcome, futile recanalization). **Table 1** indicates the demographic and clinical data of AIS patents with favorable and unfavorable 90-day functional outcomes. Age (p=0.007), frequency rate of female (p=0.021), diabetes (p=0.023) and END (p=0.002), and the NIHSS score on admission (p<0.001) were significantly higher in AIS patients who had unfavorable outcome than in those who had favorable outcome. In contrast, we did not detect statistical differences between the two groups for vascular risk factors (including hypertension, dyslipidemia, coronary artery disease, atrial fibrillation, and smoking), stroke location, stroke etiology, procedural parameters (including time from symptom onset to groin puncture, time from groin puncture to recanalization, number of passes, and intravenous thrombolysis prior to EVT), current lipid-lowering/anti-platelet/coagulation treatment, and occurrence of sICH. As shown in **Figure 4A**, there were no significant difference in the lipid mediator parameters tested in samples collected on admission between AIS patients with unfavorable outcome and those with favorable outcome (adjusted p>0.05). The plasma levels of post-EVT LXA4 (adjusted p<0.001), LXA4/LTB4 ratio (adjusted p<0.001), and ΔLXA4 (adjusted p=0.002) were significantly lower, while those of post-EVT LTB4 (adjusted p=0.015) and ΔLTB4 (adjusted p=0.036) were significantly higher in samples from patients with unfavorable outcome as compared to those with favorable outcome. However, levels of post-EVT RvD1 and RvD1/LTB4 ratio, and ΔRvD1 were comparable between the two groups (adjusted p>0.05). Furthermore, multivariate logistic regression analyses (**Figure 4B**) revealed that plasma post-EVT LXA4 levels (p=0.008) and LXA4/LTB4 ratio (p=0.024), and ΔLXA4 levels (p=0.013), but not post-EVT LTB4 (p=0.196) and ΔLTB4 were independent predictors of unfavorable 90-day functional outcome after adjustment for confounding factors (age, frequency rate of female, diabetes and END, and admission NIHSS scores). In addition, we also showed that END was an independent predictor of unfavorable 90-day functional outcome (p=0.006). As shown in **Figure 4C**, we then found that we then found that AUC for post-EVT LXA4 level and LXA4/LTB4 ratio, and ΔLXA4 level were 0.757 (95%CI: 0.5368-0.8623), 0.7399 (95%CI: 0.6317-0.8481), and 0.7209 (95%CI: 0.61-0.8319), respectively. The optimal cutoff value of post-EVT LXA4 level and LXA4/LTB4 ratio, and ΔLXA4 level were 333.8 pg/mL (sensitivity 69.77% and specificity 73.68%), 0.2452 (sensitivity 95.35% and specificity 44.74%), and 203.3 pg/mL (sensitivity 79.07% and specificity 60.53%), respectively.

### Correlations of Plasma Lipid Mediators With NLR at 24 Hrs Post-EVT

NLR reflects the severity of systemic or local disease-related inflammation, and has been validated to be a prognostic indicator (e.g., sICH, 90-day outcome, and mortality) for AIS patients treated with EVT (3, 10). We then determine the relationship between these inflammation-related lipid mediators with NLR at 24 hrs post-EVT. Pearson correlation coefficient showed that levels of anti-inflammatory/pro-resolving LXA4 (r=-0.5099, p<0.0001, **Supplemental Figure 1A**) were inversely associated with NLR, whereas levels of pro-inflammatory LTB4 (r=0.4918, p<0.0001, **Supplemental Figure 1C**) were positive associated with NLR. There was no correlation between levels of RvD1 and NLR (r=0.0413, p=0.7141, **Supplemental Figure 1B**).

## Discussion

In the present study, our main findings were that 1) post-EVT LXA4 level, LTB4 level, the LXA4/LTB4 and RvD1/LTB4 ratios, ΔLXA4 level, and ΔLTB4 level were independent predictors for END, 2) post-EVT LXA4 level and LXA4/LTB4 ratio, and ΔLXA4 level were independent predictors for unfavorable 90-day functional outcome in patients with AIS who treated with successful EVT. However, the question remains as whether these variables are the cause or the result of the futile recanalization, which need further studies to determine the causal-effect relationship.

Several generic markers including age, gender, stroke severity, time from onset to puncture, and comorbidities (hypertension, diabetes, and atrial fibrillation) have been reported associated with clinical outcomes after EVT (4, 7, 22). In the present study, we also identified that the unfavorable functional outcome group was older, and had higher proportion of female, higher incidence of diabetes mellitus, and higher baseline NIHSS scores. However, the predictive values of these generic markers are poor and often non-specific. In addition, we also found that END was an independent predictor of unfavorable functional outcome, which was consistent with previous studies (23). Several recent studies have found that EVT procedural-related factors (e.g., first-pass effect versus multiple-pass effect) and post-procedural complications are strong predictors of unfavorable functional outcome (24, 25). In addition, radiological markers are emerging, CT-based texture, blood-brain barrier disruption, brain atrophy, and leukoaraiosis, CT perfusion-based cerebral perfusion parameters, and diffusion-weighted imaging (DWI)-based large deep white-matter lesions have demonstrated to predict prognosis after EVT (26–30). Moreover, blood-based biomarkers are also found to be predictive for unfavorable early (END) and long-term functional outcomes of EVT-treated AIS patients, including interleukin-6, NLR, N-terminal probrain natriuretic peptide, albumin, matrix metalloprotease-9, enascin-c, thioredoxin, ADAMTS-13 (a disintegrin and metalloproteinase with a thrombospondin type 1 motif, member 13, and occludin, etc. (2, 3, 10, 31–35). We have recently reported that high levels of the pro-resolving protein mediator annexin A1 correlated with 3-month clinical outcomes of AIS patients who underwent successful recanalization by EVT (36). However, none of these biomarkers has been sufficiently reliable for guidance in clinical practice, which still need to be validated in large clinical trials. Meanwhile, it is urgent to further explore the recanalization by EVT-induced cellular, biochemical, and molecular changes, and identify more novel biomarkers.

Neuroinflammatory responses, characterized by glial activation, recruitment of peripheral immune cells, and production of inflammatory mediators have been implicated in the pathophysiology of cerebral I/R injury at multiple stages. Effective resolution of excessive and uncontrolled inflammation is essential for balancing cerebral I/R-induced neuroinflammation to restore brain homeostasis and to avoid neurological impairments (8–10). For example, cells subjected to brain ischemia accumulate PUFAs and their metabolites lipid mediators, which regulate both the pro-inflammatory and the anti-inflammatory/pro-resolving processes (12). After the onset of AIS, PUFAs are released from the cellular membrane by the activities of phospholipase enzymes (e.g., phospholipase A2), pro-inflammatory classical eicosanoids, including prostaglandins and leukotrienes, are then produced from AA and promote the resident microglial activation, neutrophil infiltration, and vascular permeability. Next, prostaglandins, primarily PGE2, initiate the lipid mediator class switch *via* activating the biosynthesis of the SPMs from both n-6 PUFA AA and n-3 PUFAs DHA and EPA. SPMs actively resolute inflammation to homeostasis by limiting neutrophil activation and recruitment, decreasing the production of pro-inflammatory mediators, increasing the production of anti-inflammatory mediators, and enhancing non-phagocytic recruitment and phagocytosis (efferocytosis) of macrophages (14–16). These bioactive lipid mediators may therefore represent as predictive markers of stroke severity as well as the outcomes, and serve as potential adjuvant therapeutics. However, these theories are largely based on experimental evidence, few clinical investigations of lipid mediators in patients with AIS have been conducted so far.

The pro-inflammatory LTB4 is synthesized from AA through 5-lipoxygenase (5-LOX). Upon binding to its B-leukotriene receptor (BLTR, including BLT1 and BLT2), LTB4 promotes the activation and chemotaxis of inflammatory cells (mainly neutrophil) and enhances the production of other pro-inflammatory mediators (37). Neutrophils are a major source of LTB4 accumulated in cells of the ischemic brains and circulation during acute stroke (38). LTB4 administration increased infarct volume, while strategies reducing LTB4 production (5-LOX inhibitor) and/or inhibiting its receptor (BLTR antagonism) could exert neuroprotective effects against cerebral I/R injury (38–40). Chan et al. (38) reported that levels of plasma LTB4 in AIS patients with MCA occlusion were increased rapidly, reaching its peak level on day 1 from symptom onset to almost twice of the initial post-stroke levels, and that higher levels of LTB4 on days 0 and 7 are associated with poor 3-month clinical outcomes. Similar to these findings, we found that LTB4 levels were significantly increased after successful recanalization, and the increased post-EVT LTB4 and ΔLTB4 levels were inversely associated with 3-month clinical outcome. Furthermore, AIS patients with unfavorable early (END) and long-term functional outcomes had higher levels of post-EVT LTB4 and ΔLTB4 than those with favorable outcome. After adjusting for confounding factors, these two variables were proven served as independent predictors for END but not 90-day functional outcome, which may be most likely caused by too small sample size. These results indicated that reperfusion after EVT provoke excess and uncontrolled neuroinflammatory responses that may ultimately lead to early and permanent neurological deficits and poor prognosis of AIS patients.

LXA4 is the first described and most intensively studied SPM that is generated from AA *via* LOX-mediated transcellular biosynthesis (41, 42). Pre-clinical researches in atherosclerotic plaque formation, vulnerable plaque rupture, occurrence of AIS, and post-stroke recovery have confirmed that LXA4 or its analogs resolute acute inflammation and prevent chronic inflammation by activating formyl peptide receptor type 2/LXA4 receptor (FPR2/ALX) expressed on various target cells (e.g., endothelial cells, leukocytes, and microglia/macrophages). The potential mechanisms include preventing BBB disruption, reducing leukocyte and platelet activation, and neutrophil-platelet aggregate (NPA) formation, inhibiting microglial activation, promoting microglia/macrophage polarization to anti-inflammatory M2 type, decreasing the release of pro-inflammatory cytokines, and increasing the release of anti-inflammatory cytokines (9, 43–47). In addition, LXA4 has demonstrated to reverse cholesterol transport, decrease levels of low-density lipoprotein (LDL), reduce oxidized LDL-induced inflammation, and inhibit foam cell formation, thus contributing to plaque regression (48, 49). Wang et al. (17) reported that circulating LXA4 is related to cognitive status in ischemic stroke patients, and decreased LXA4 are associated with post-stroke cognitive impairment (PSCI). Consistent with these reports, we found that the LXA4 levels were reduced during the onset of AIS but significantly increased after the successful recanalization by EVT. The increased post-EVT levels of LXA4 and ΔLXA4 were more pronounced in AIS patients with favorable functional outcome. Finally, we found that these two variables independently predicted both early (END) and long-term outcomes after adjusting for confounding factors. These results indicated that cerebral ischemia impaired resolution of inflammation and that anti-inflammatory/pro-resolving mechanisms are activated immediately after recanalization. Notably, NPA has been proven to be a major source of LXA4 (50, 51). Considering cerebral I/R injury is accompanied by neutrophil and platelet activation, and NPA formation within cerebral microvessels (9), increased LXA4 may exert crucial neuroprotective roles in inhibiting excessive acute inflammatory response after EVT treatment, and inadequate production of LXA4 contribute to cerebral I/R injury, and subsequent END and futile recanalization, but their underlying mechanism remains poorly understood.

RvDs are first identified in brain tissues, and they are synthesized from DHA. Studies indicated that RvDs are biosynthesized *via* transcellular biosynthesis with interactions between neutrophil and hypoxic vascular endothelial cells (52–54). RvD1 is most intensively studied RvDs, and exerts its potent anti-inflammatory and pro-resolving properties mediated through ALX/FPR2 or D resolving receptor 1 (DRV1/GPR32) in neurological diseases (55, 56). RvD1 is reported to promote atherosclerotic plaque rupture, and delivery of RvD1 in animal models produced a beneficial effect on plaque stability (19, 57). The levels of plasma RvD1 are lower in patients presenting acute plaque rupture events than those in patients with asymptomatic carotid disease (58). Szczuko et al. (59) reported that plasma RvD1 levels in patients with AIS were significantly lower in comparison to control subjects, and suggested that RvD1 is one of the most important PUFA derivatives in reducing post-stroke inflammation. Kotlęga et al. (60) demonstrated that higher levels of RvD1 were associated with better cognitive functions in the acute phase of stroke and during the 6-month follow-up. In an experimental model of cerebral I/R injury, RvD1 could reduce oxidative stress, inflammatory response, and neuronal apoptosis, probably by inactivating NLRP3 (NOD-like receptor family pyrin domain containing 3) inflammasome (61). In addition, the RvD1 precursor DHA prevents retrograde amnesia by changing acute cerebral I/R injury rather than stimulating memory performance (62, 63). Similar with LXA4, we found that plasma levels of RvD1 decreased during AIS onset but increased significantly post-EVT. However, we did not find association between RvD1 or ΔRvD1 and clinical outcomes, indicating that RvD1 may not play a decisive role in acute phase of post-EVT cerebral I/R injury.

The dynamic balance between pro-inflammatory lipid mediators and SPMs during acute inflammation defines the duration and intensity of inflammatory responses and the timing of tissue resolution (19). As such, their imbalance can lead to chronic inflammation and tissue injury. The ratio of SPMs to LTB4 may indicate the balance state between pro-resolving and pro-inflammatory signals. Wang et al. (17) demonstrated that the reduction of LXA4 to LTB4 ratio was inversely correlated with PSCI. Miao et al. (18) found that diabetes mellitus impairs the process of inflammation resolution in AIS, and the ratio of RvD2/LTB4 was correlated with the prognosis of acute AIS. In atherosclerosis, a reduced RvD1 to LTB4 ratio measured in salivary fluids predicts the thicker intima media of the carotid artery (64). Moreover, there is a decreased ratio of RvD1 to LTB4 in the tissues collected from vulnerable atherosclerotic plaques (19). In the present study, we showed that higher LXA4/LTB4 ratio independently predicted better early and long-term outcomes of the AIS patients, and the RvD1/LTB4 ratio independently predicted END. The latter may be due to significant differences in LTB4. In addition, we also found LXA4 were inversely, while LTB4 were positively associated with systemic/local inflammatory state (evaluated by NLR). Taken together, combined with experimental researches on mechanisms, our study suggests that the imbalance between the pro-resolving/anti-inflammatory lipid mediator LXA4 and the pro-inflammatory LTB4 may weaken the neuroinflammation resolution, thus leading to post-EVT cerebral I/R injury and chronic inflammation progresses.

This study has several limitations. First, our data provides a basis for speculation that lipid mediator could be an important mechanism underlying reperfusion injury, but these data cannot establish a causal-effect relationship. Second, this is a retrospective study, and the relatively small sample size may weaken the statistical power of detecting subtle differences. Third, we adjusted confounding factors that achieved statistical difference in the multivariate logistic regression analysis. There may be additional confounding factors that could influence the conclusion. Fourth, we collected blood samples at 24 hrs post-EVT, potentially insufficient to detect the dynamic changes of the lipid mediator-mediated inflammation to better predict clinical outcomes. Finally, the study may require validation in independent patient cohorts and using the state-of-art technologies such as liquid chromatography-mass spectrometry, which has been extensively used in quantifying small molecules such as lipid mediators in biological samples. Our study serves as a pilot and exploratory study that lays the foundation for large, sufficiently powered, and prospective multicenter trials.

## Conclusions

An imbalance between the pro-resolving/anti-inflammatory lipid mediator LXA4 and the pro-inflammatory LTB4 developed during the acute phase of AIS patients with first-ever LVO who underwent complete angiographic recanalization by EVT. Plasma levels of lipid mediators were independent predictors for early and long-term functional outcomes. Additional studies are needed to investigate the causal-effect relationship, and whether targeting inflammation-related lipid mediators has therapeutic potential.

## Data Availability Statement

The original contributions presented in the study are included in the article/**Supplementary Material**. Further inquiries can be directed to the corresponding authors.

## Ethics Statement

The studies involving human participants were reviewed and approved by Ethics Committees of Liaocheng People’s Hospital and Xuanwu Hospital, Capital Medical University. The patients/participants provided their written informed consent to participate in this study.

## Author Contributions

LZ, LJ, XX, and JH conceived and designed the study. YFe, XX, and WG developed methodology. JH, YFe, XX, MZ, YFa, TY, and BY carried out the experiments. KY performed data analysis. XX, KY, BY, and WG interpreted the results and prepared the figures and tables. GD and JW provided technical support. XX and JH drafted the manuscript. LZ and LJ reviewed and revised the manuscript and supervised the study. All authors read and approved the final manuscript. All authors contributed to the article and approved the submitted version.

## Funding

This research was supported by grants from the National Natural Science Foundation of China (grant 82001317, 82171303, and 81801231), the National Key Research & Development Project (grant 2016YFC1301703).

## Conflict of Interest

The authors declare that the research was conducted in the absence of any commercial or financial relationships that could be construed as a potential conflict of interest.

## Publisher’s Note

All claims expressed in this article are solely those of the authors and do not necessarily represent those of their affiliated organizations, or those of the publisher, the editors and the reviewers. Any product that may be evaluated in this article, or claim that may be made by its manufacturer, is not guaranteed or endorsed by the publisher.
