# Supplementary material for: Plasma Lipid Mediators Associate With Clinical Outcome After Successful Endovascular Thrombectomy in Patients With Acute Ischemic Stroke
Source: Front Immunol. 2022 Jul 4;13:917974. doi: 10.3389/fimmu.2022.917974 (PMC9295711; doi:10.3389/fimmu.2022.917974)
Supplement: Supplementary file 1 [file DataSheet_1.docx]

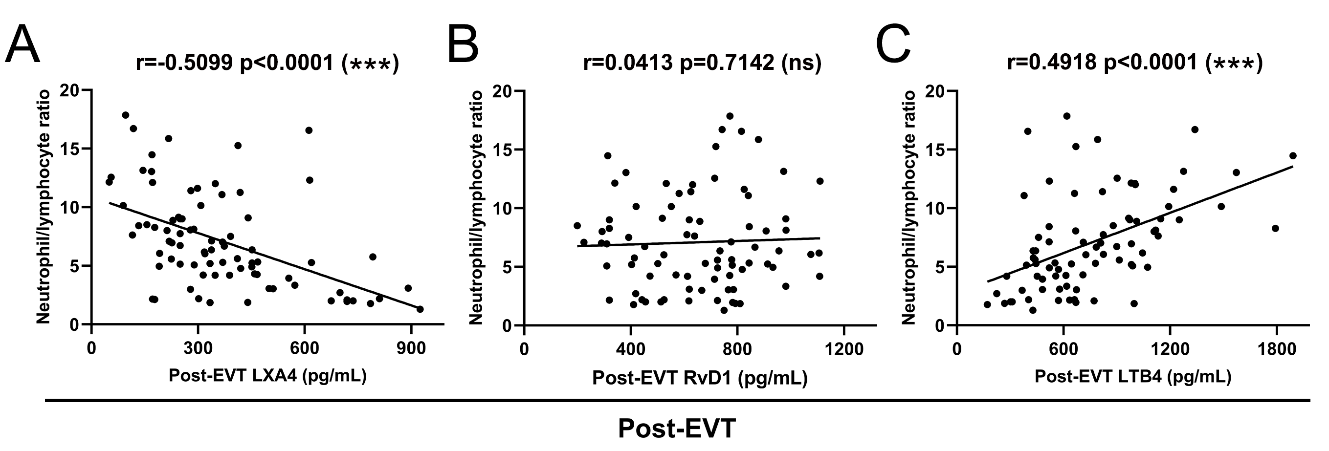


**Supplemental Figure 1 Pearson correlation coefficient analyses of correlations of plasma lipid mediators with neutrophil/lymphocyte ratio (NRL) at 24 hrs post-EVT.**
